# Application of Eye Tracking Technology in Medicine: A Bibliometric Analysis

**DOI:** 10.3390/vision5040056

**Published:** 2021-11-11

**Authors:** Gianpaolo Zammarchi, Claudio Conversano

**Affiliations:** Department of Economics and Business Sciences, University of Cagliari, 09123 Cagliari, Italy; conversa@unica.it

**Keywords:** eye tracking, gaze tracking, autism spectrum disorders, psychiatric disorders, eye movements

## Abstract

Eye tracking provides a quantitative measure of eye movements during different activities. We report the results from a bibliometric analysis to investigate trends in eye tracking research applied to the study of different medical conditions. We conducted a search on the Web of Science Core Collection (WoS) database and analyzed the dataset of 2456 retrieved articles using VOSviewer and the Bibliometrix R package. The most represented area was psychiatry (503, 20.5%) followed by neuroscience (465, 18.9%) and psychology developmental (337, 13.7%). The annual scientific production growth was 11.14% and showed exponential growth with three main peaks in 2011, 2015 and 2017. Extensive collaboration networks were identified between the three countries with the highest scientific production, the USA (35.3%), the UK (9.5%) and Germany (7.3%). Based on term co-occurrence maps and analyses of sources of articles, we identified autism spectrum disorders as the most investigated condition and conducted specific analyses on 638 articles related to this topic which showed an annual scientific production growth of 16.52%. The majority of studies focused on autism used eye tracking to investigate gaze patterns with regards to stimuli related to social interaction. Our analysis highlights the widespread and increasing use of eye tracking in the study of different neurological and psychiatric conditions.

## 1. Introduction

Eye tracking is a technique used to measure and study the range of eye movements of participants while they are engaged in different activities (e.g., during reading, assessment of a visual stimulus and so on), in a non-invasive way and with a high degree of precision. The assessment of eye movements is made through the eye tracker, a device that sends out a beam of invisible near-infrared light that is reflected in the cornea. After the reflection is collected by the eye tracker’s sensors, it is possible to apply algorithms to calculate where a person is looking. An eye tracker can capture the position of the eyes several times per second. It is, therefore, possible to produce a visual map to measure how and for how long the person looked at different visual stimuli. In addition to being useful for several commercial applications (e.g., to assess the impact of different aspects of packaging and/or to improve their visual presentation [1,2], evaluate the usability of websites [3] and so on), eye tracking has been increasingly applied to the study of different medical conditions, such as neurological and psychiatric disorders, based on the observation that eye movement can provide insights into cognitive processing [4]. 

Considerable evidence suggests that patients with different psychiatric and neurological conditions show abnormalities in eye movement. For instance, autism spectrum disorders (a group of neurodevelopmental disorders characterized by repetitive behaviors and alterations in social interaction/communication, language and nonverbal communication [5]) are associated with alterations in gaze patterns when exposed to different types of stimuli [6]. Moreover, patients with schizophrenia or bipolar disorder show distinct patterns of eye movement during smooth pursuit and visual search [7]. While studies on eye movement characteristics in these disorders have been conducted since the early 1900s, the increasing accessibility of eye tracking technology has made it possible to provide precise and quantitative measurements of these impairments. In the last few years, an increasing number of studies have investigated gaze behavior in individuals with different psychiatric disorders besides those already mentioned (for instance, eating disorders [8]) or with neurological disorders. For instance, eye tracking has been applied to the study of stroke [9], brain injury [10] or neurodegenerative disorders such as Alzheimer’s disorder or Parkinson’s disorder [11,12].

The eye tracking technique produces objective data, which are not influenced by the opinions of the subjects carrying out the study or by those who analyze the results. Moreover, the eye tracker can also be used in combination with other neurophysiological or brain imaging techniques, such as electroencephalography (EEG) or magnetic resonance imaging (MRI), to increase the understanding of the underlying neurobiological processes. Overall, eye tracking represents a non-invasive technique to collect objective and precise data allowing for the study of complex phenotypes such as cognition, emotion and social interaction.

In this article, we provide a comprehensive and up-to-date bibliometric analysis of studies that applied eye tracking technology to different medical fields, aiming to describe trends regarding the most investigated conditions and to identify countries and institutions with the highest scientific production and map collaboration networks. Our analysis fills a gap in the literature by providing novel insights on the current trends as well as emerging and declining themes in the application of eye tracking to the study of different medical conditions.

## 2. Materials and Methods

### 2.1. Literature Search

We conducted a literature search on the Web of Science Core Collection (WoS) online database updated to the 29 August 2021 (the day on which the study records were downloaded from WoS) to identify scientific articles or proceeding papers that used eye tracking to study different disorders. The search strategy was the following: (“eye track*” OR “eye-track*” OR “gaze track*” OR “gaze-track*”) AND (disorder* OR disease*). We searched for studies mentioning these terms in any searchable field (default option in WoS). No language or date restrictions were applied. As suggested in the Bibliometrix documentation, we used WoS instead of Scopus, as the former is preferable in terms of data quality for a bibliometric search due to the standardization of cited reference items, for example. In addition, WoS and not Scopus allowed us to split the collection of records retrieved with the search into multiple downloads. 

### 2.2. Bibliometric Analysis

From each identified article, the following characteristics were extracted and used for the bibliometric analysis: title, abstract, keywords, authors’ affiliations, year of publication, journal title and the number of citations. We used the freely available VOSviewer software to conduct a term co-occurrence analysis based on the network of scientific publications retrieved with the literature search and to plot the network map [13]. Terms were extracted from the titles and abstracts of the retrieved articles using the default options in VOSviewer (binary counting method and minimum of ten occurrences for included terms). In the co-occurrence term map based on network data, circles represented terms, the size of the circle was proportional to the number of articles in which the term was found and the curved lines between circles represented the strength of the relatedness of two terms (i.e., terms with a high number of co-occurrences were linked by a thicker line). Terms strongly related to each other formed clusters of nodes that were identified based on the modularity-based clustering algorithm implemented in VOSviewer [13] and represented using different colors. The VOSviewer software allowed us to highlight the relationship between two or more nodes, creating a distance-based map of terms. For each node, the VOS clustering techniques first compute a normalized association strength, then place nodes on a bi-dimensional plane and lastly assign them to a cluster (i.e., a group of closely related nodes). When a network is represented, it is normal for some nodes to have much more connections than others. If normalization is not applied, the disparity between nodes with different numbers of edges, i.e., nodes with a high number of edges and a node with a small number of edges, would make it difficult to represent the network effectively. VOSviewer applies the normalization criterion proposed by Van Eck and Waltman [14]. Let *a_ij_* be the weight of the edge between node *i* and node *j*, which might be equal to zero in the case of no connection. The normalization association strength allows us to create a network where the weight of the edge between nodes *i* and *j* is given by:

(1) $$s_{ij}=\frac{2ma_{ij}}{k_{i}k_{j}}$$

where *k_i_* and *k_j_* are the sum of the weights of all the edges of node *i* and node *j*, and *m* is the sum of the weights for all edges in the network. 

After computing the weights for all nodes, a variant of the Multidimensional Scaling Using Majorization (SMACOF) algorithm [15] is applied to minimize

(2) $$V\left(x_{1},\ldots ,x_{n}\right)=\sum^{\text{​}}s_{ij}{\left|⃒x_{i}-x_{j}⃒\right|}^{2}$$

subject to the constraint

(3) $$\frac{2}{n\left(n-1\right)}\sum^{\text{​}}\left|⃒x_{i}-x_{j}⃒\right|=1$$

where *n* is the number of the network’s nodes and $\left|⃒x_{i}-x_{j}⃒\right|$ is the Euclidean distance between nodes *i* and node *j*. In this way, we could compute the distances between each node and represent the network in a bi-dimensional space where, for each pair of nodes, their distance is proportional to the strength of their relationship [16]. Finally, each node is assigned to one cluster [17] using a variant of the modularity function introduced by Newman and Girvan [18] and Newman [19]:

(4) $$V\left(c_{1},\ldots ,c_{n}\right)=\sum^{\text{​}}\delta \left(c_{i},c_{j}\right)\left(s_{ij}-\gamma \right)$$

where $c_{i}$ is the cluster to which node *I* is assigned, $\delta \left(c_{i},c_{j}\right)$ is an indicator function that equals 1 only when $c_{i}=c_{j}$*,* and γ is a parameter that determines the number of clusters (larger values of γ means more clusters).

Next, we used the Bibliometrix package [20] version 3.1 in R version 4.1.1. [21] and the Biblioshiny shiny app [20] to conduct bibliometric analyses on our dataset. We computed the annual growth rate and identified peaks based on the year of publication of the retrieved articles. We identified countries with the highest number of published articles and/or citations (based on the affiliation of corresponding authors as well as of other authors) and plotted the country collaboration network. The country collaboration network was generated using the Louvain clustering algorithm implemented in Bibliometrix, setting the number of nodes to 20 for better clarity of representation. We also identified the most influential institutions based on the number of retrieved articles. A word cloud of the most frequent keywords was plotted using the wordcloud2 package in R [22]. Scripts developed to conduct the described analysis are available at [23]. Finally, we used Bibliometrix to analyze the evolution of themes investigated in the retrieved articles based on a clustering analysis of keywords. To this aim, we divided the timespan into three periods identified based on the computed annual growth rate, and for each period we constructed a thematic map. In this map, each cluster was represented with a bubble. The horizontal axis represented Callon’s centrality (i.e., the importance of the theme in the research field) while the vertical axis represented Callon’s density (a measure of the theme’s development) [24].

Based on the results of the previously described analyses (frequency of keywords related to this disorder, sources of retrieved articles as well as the thematic evolution analysis) we identified autism spectrum disorders as the most investigated theme. Therefore, to conduct an in-depth analysis on this topic, we repeated the analyses on a subgroup of 638 articles focused on autism spectrum disorders. 

## 3. Results

### 3.1. Trends Regarding Most Investigated Disorders 

**Figure 1:**
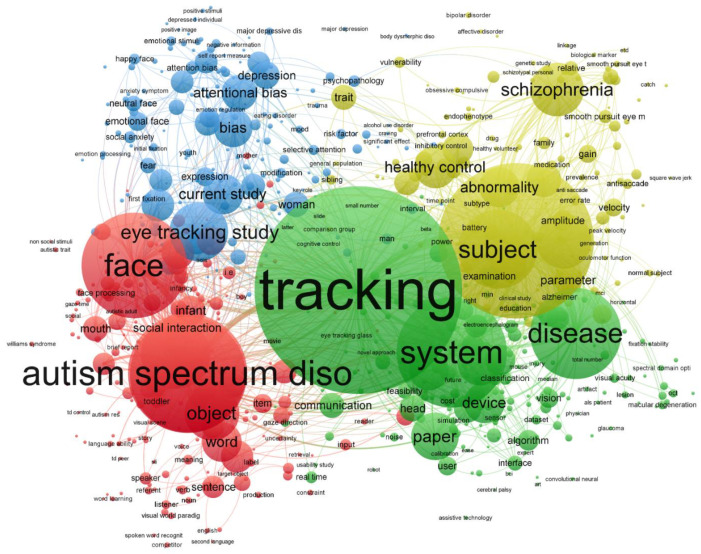
Co-occurrence term map of studies investigating eye tracking in different disorders.

A total of 2456 documents (2141 journal articles and 215 proceeding papers), published from 1991 up to 29 August 2021 were retrieved. The analysis of research fields conducted using the WOS analytical instrument showed that the most represented area was psychiatry (503, 20.5%) followed by neuroscience (465, 18.9%) and psychology development (337, 13.7%). The annual scientific production growth was 11.14% and showed exponential growth with three main peaks in 2011, 2015 and 2017 (Figure S1). Figure 1 shows a term co-occurrence network in which circles represent terms, the size of the circle is proportional to the number of articles in which the term was retrieved and the thickness of the lines between circles represents the strength of the relatedness of two terms. Four main clusters of terms strongly related to each other were identified by VOSviewer: (1) red, focused on autism spectrum disorders, (2) blue, including studies investigating attentional bias, emotion recognition, major depressive disorder and anxiety disorders; (3) yellow, including studies on schizophrenia, bipolar disorder and obsessive-compulsive disorder, and (4) green, including more generic terms (e.g., tracking, disease) as well as terms related to disorders such as cerebral palsy or amyotrophic lateral sclerosis (ALS). 

**Figure 2:**
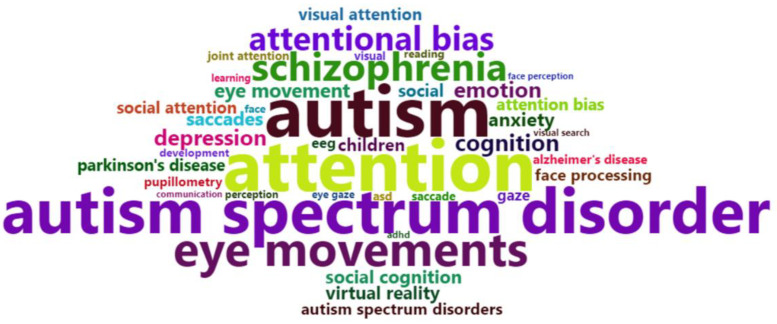
Word cloud based on the most frequent keywords of articles retrieved with the search. The size of the words is proportional to their frequency.

A word cloud plotted using the most frequent keywords (after the exclusion of words included in the search strategy), is shown in Figure 2. As in the co-occurrence term map, autism spectrum disorders were the most represented of the conditions, followed by schizophrenia, depression, anxiety, Parkinson’s disease and Alzheimer’s disease. 

### 3.2. Countries and Institutions 

Studies were conducted by investigators from 2204 institutions located in 63 countries. The three countries with the highest scientific production in terms of the retrieved articles, based on the affiliation of the corresponding author, were the USA (867, 35.3%), the UK (234, 9.5%) and Germany (178, 7.3%) (Table 1). 

**Figure 3:**
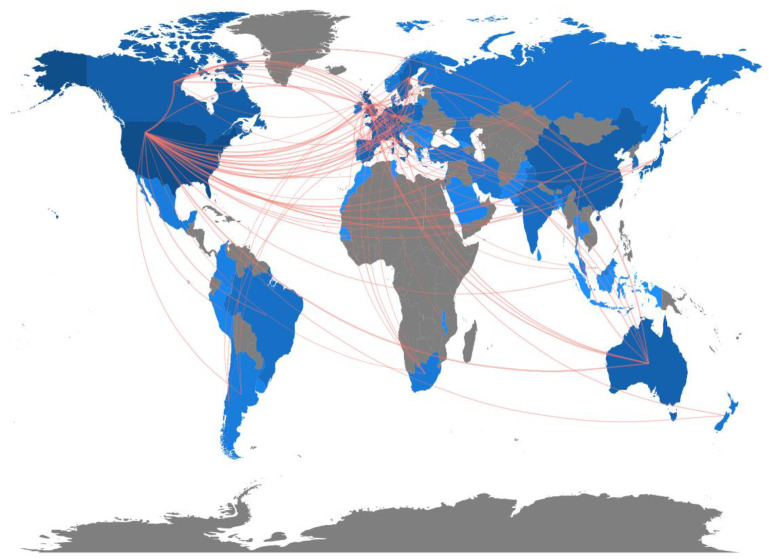
Map of international scientific collaborations for studies investigating eye tracking in different disorders. The color intensity is proportional to the number of international collaborations (gray represents the lack of collaborations retrieved in our search).

A map of international collaboration based on the affiliations of authors is shown in Figure 3. The width of the lines is proportional to the number of studies in which a collaboration between two countries was observed. The USA showed the highest number of collaborations (42 countries) followed by the UK (33 countries) and Germany (20 countries). Collaborations present in the highest number of articles were those between the USA and Canada (47 articles), the UK (47 articles), China (31 articles) and Germany (29 articles), highlighting strong collaborations between the countries with the highest scientific production identified in our search (Table S1). However, the USA showed a modest collaboration percentage (calculated as the ratio between articles with international collaborations and articles with no international collaborations, multiplied by 100), as only 15% of articles attributed to the USA based on the affiliation of the corresponding author included international collaborations (Table 1). Among countries with at least ten published articles, Finland showed the highest collaboration percentage, while Japan showed the lowest (Table 1). For most countries, the international collaboration percentage was approximately 30% (one-third of studies included international collaborations).

**Figure 4:**
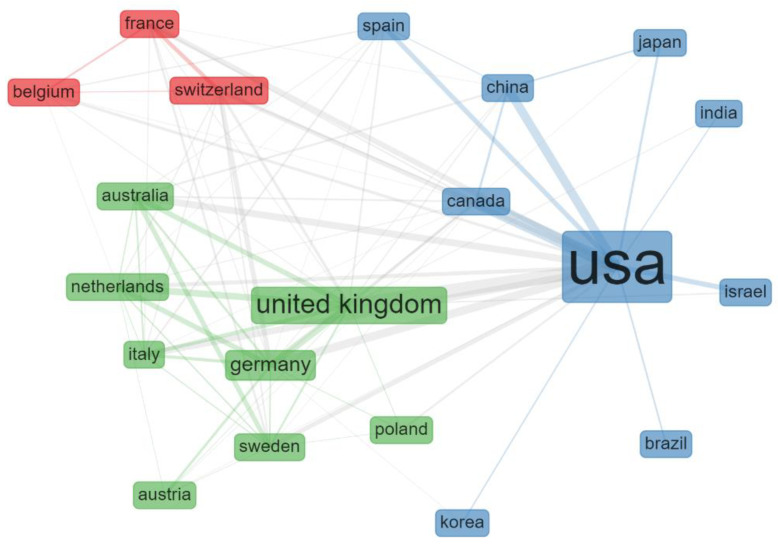
Country collaboration network of articles retrieved in the search. The size of the country’s name is proportional to the number of articles retrieved for that country.

A country collaboration network was generated based on the approach proposed by previous articles [20,25]. We identified three main clusters, two of which were centered on the two countries with the highest scientific production (USA and UK). A third smaller cluster included France, Belgium and Switzerland (Figure 4). 

The USA also received the highest number of citations (23,434), while the highest average number of citations per article was received by Singapore (38.50), Canada (35.90) and Norway (35.71) (Table S2). Among institutions, the University of Toronto ranked first for the number of articles (88), followed by Vanderbilt University in Nashville (83) and the University of California in Davis (81) (Table S3).

Of the three most relevant sources for the retrieved articles, two were related to autism spectrum disorders: the Journal of Autism and Developmental Disorders (104 articles), PLOS ONE (65 articles) and Autism Research (51 articles) (see the list of journals in which at least ten articles were published in Table S4). 

### 3.3. Trends in Topics

**Figure 5:**
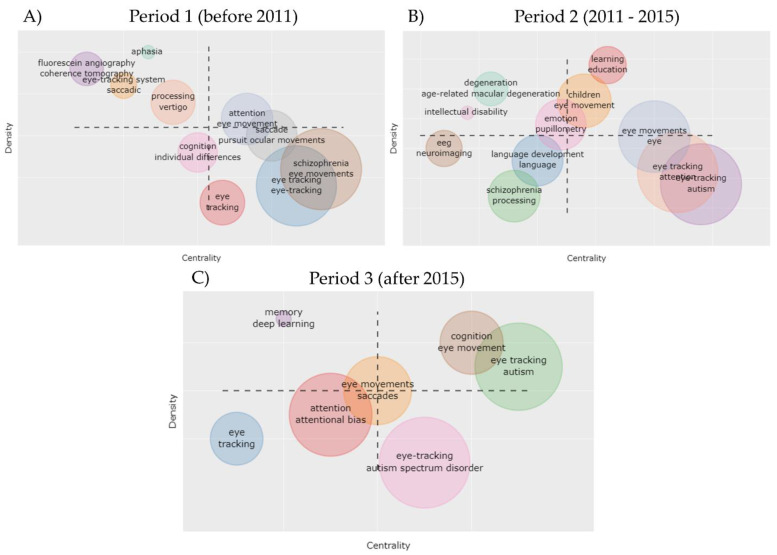
Thematic evolution of eye tracking studies. A thematic map is constructed via the application of a clustering algorithm on the network of keywords of the retrieved articles. Thematic networks are plotted in two dimensions, where axes are the function of centrality (the importance of the theme in the research field, horizontal axis) and density (a measure of the theme’s development, vertical axis) of the thematic network. Three time slices are represented: (**A**) Period 1: before 2011; (**B**) Period 2: 2011–2015; and (**C**) Period 3: after 2015. For each period, the left upper quadrant (high density and low centrality) shows highly developed and isolated themes (niches), the right upper quadrant (high density and high centrality) shows motor themes, the left lower quadrant (low density and low centrality) shows emerging or declining themes and the right lower quadrant (low density and high centrality) shows basic and transversal themes. Each bubble represents a cluster of topics (colors indicate different clusters). The name of the two topics with higher occurrence is shown in the bubble. The size of the bubble is proportional to the cluster word occurrences, while the bubble position is set according to the cluster centrality and density.

To identify trends in topics, such as emerging or declining themes, we generated a thematic map, which is a Cartesian representation of the thematic clusters identified by performing a cluster analysis on a co-occurrence network of keywords. In order to study the evolution of themes, we divided the timespan into three periods based on two of the peaks previously identified through the analysis of the scientific growth of eye tracking (Figure S1) and plotted a thematic map for each period (Figure 5). In the thematic map, each bubble represents a cluster of keywords, the horizontal axis represents Callon’s centrality (i.e., the importance of the theme in the research field) while the vertical axis represents Callon’s density (a measure of the theme’s development) [24]. The left upper quadrant (high density and low centrality) shows highly developed and isolated themes (niches); the right upper quadrant (high density and high centrality) shows motor themes; the left lower quadrant (low density and low centrality) shows emerging or declining themes, and the right lower quadrant (low density and high centrality) shows themes transversal to different research areas in the field. The topics that during the time periods moved from the left lower quadrant to the right quadrants, represent topics that are moving towards mainstream theme areas, while topics moving from the right to left quadrants (i.e., topics with decreasing centrality) represent declining themes. In the first period (Figure 5A), we identified four niche themes, two of which represented the medical conditions of aphasia and vertigo. Transversal themes included pursuit ocular movements and the psychiatric disorder schizophrenia. In the second period (Figure 5B), schizophrenia moved to the left lower quadrant, suggesting a loss of centrality for this theme. Niche themes for this period included age-related macular degeneration and intellectual disability. Learning/education was identified as a motor theme and the neurodevelopmental disorder autism was found to represent a basic and transversal theme. Finally, in the third period (Figure 5C), autism was included among transversal and motor themes, while deep learning was identified as a niche theme. This finding suggests that recent studies are increasingly applying machine learning methods to eye tracking data, exploring the possibility of using eye movements as potential diagnostic markers to discriminate between patients and controls or as biomarkers of response to different interventions. 

### 3.4. Case Study: Autism Spectrum Disorders

Based on results from the analyses regarding the co-term occurrence and the most frequent keywords and sources of retrieved articles, we identified autism spectrum disorders as the most investigated condition for which eye tracking was applied. We, therefore, conducted a bibliometric analysis limited to articles focused on this topic. To this aim, among the 2456 articles retrieved with our search, we extracted the 638 articles that mentioned the words “autism” or “autism spectrum disorders” or “ASD” in the title or abstract fields. These documents included 584 articles and 54 proceedings papers published from 2002 to 2021. 

The annual scientific production growth was 16.52% and showed exponential growth with three main peaks in 2011, 2014 and 2018 (Figure S2). Studies were conducted by researchers affiliated with 781 institutions located in 37 countries. The three countries with the highest scientific production in terms of retrieved articles, based on the affiliation of the corresponding author, were the USA (234, 36.7%), the UK (88, 13.8%) and Germany (52, 8.2%) (Table 2). 

The USA showed the highest number of collaborations (22) followed by the UK (20), France, Italy and Switzerland (10). Collaborations present in the highest number of articles were between the USA and China (17 articles), the UK (15 articles) and Canada (15 articles) (Table S5). Nonetheless, most articles attributed to the USA (based on the affiliation of the corresponding author) did not include authors from other countries. Among countries with at least ten published articles, Switzerland showed the highest collaboration percentage, while Japan showed the lowest as in the original search (Table 2). Most of the countries showed a percentage of international collaboration of approximately 30% (approximately one-third of studies included international collaborations).

The country collaboration network shows four clusters (Figure S3). In addition to two main clusters centered on the USA and UK (as in the original search), we identified two small clusters including France–Switzerland and Sweden–Australia (Figure S3). The USA also received the highest number of citations (7671), while the highest average number of citations per article was received by Singapore (71.50), the Netherlands (34.91) and Denmark (33.33) (Table S6). The most relevant affiliations for the number of articles were Vanderbilt University in Nashville (54), La Trobe University in Melbourne (45) and Duke University in Durham (44) (Table S7). 

Table 3 shows that the articles with the highest number of citations are mainly focused on the analysis of face processing and social interactions. The majority of these articles investigated differences in eye movements during the observation of visual stimuli related to social cues or social interactions, such as images or videos depicting faces [26,27,28,29,30,31,32,33,34,35,36,37,38] or social scenes/interactions [39,40,41,42,43,44]. 

## 4. Discussion

In this article, we conducted a bibliometric analysis of studies investigating eye tracking in different medical conditions. We observed a substantial growth of the use of this technology in the last few years and identified strong collaboration networks among the countries with the highest scientific production (USA, UK and Germany). This substantial growth might be explained by the increased availability of eye tracking systems at different price ranges as well as by open-source data acquisition software. Based on the most frequent keywords, autism spectrum disorders represented the most investigated condition, followed by other psychiatric disorders (schizophrenia and depression) and neurodegenerative disorders (Parkinson’s disease and Alzheimer’s disease). Different causes might explain the observed relevance of eye tracking technology for research on autism spectrum disorders. First, autism represents a neurodevelopmental disorder and eye tracking is a non-invasive technique that can be applied to studies including infants and children. Second, different studies support the hypothesis that patients with this condition focus their attention on different types of stimuli compared to typically developing individuals, especially in the case of stimuli related to social interactions. Indeed, an analysis of the most cited articles retrieved in our search and related to autism identified social interactions and face processing as the most investigated field. The majority of these articles used eye tracking to investigate differences in gaze patterns during vision exposure to social stimuli, such as faces or social events, either in patients with autism [27,28,29,30,31,32,33,34,35,37,38,40,41,42,43], in their siblings [26,36] or participants at risk of autism [39] compared with typically developing controls. Some of these studies combined eye tracking with additional techniques such as MRI in order to investigate whether the identified differences in gaze patterns were associated with neurobiological abnormalities [26,27,45]. In the most cited article, Dalton and colleagues showed that patients with autism spent less time looking at the eye region when presented with photographs of human faces compared to typically developing controls [27]. The authors showed that the amount of time spent observing the eye region positively correlated with the activation of a specific brain area (fusiform gyrus), measured with MRI. In a subsequent study, the authors also showed similar findings in unaffected siblings of patients with autism spectrum disorders compared with controls [26]. Abnormalities identified using eye tracking might also be associated with the increased severity of illness as shown by Jones and colleagues in a study including 2-year-old children with autism [28]. In this study, children with autism spent less time looking at the eyes and more time looking at the mouths of people pictured in videos compared with typically developing children or children with a developmental delay different from autism. Importantly, children with autism showing a low number of fixations located on eyes also showed higher levels of social disability [28]. 

Our analysis of trend topics supported the relevance of autism spectrum disorders as a central theme and identified “deep learning” as the niche theme that is mostly reported in recent articles. This finding might be explained by the fact that, in the last few years, a number of studies have started to explore the utility of eye tracking markers to improve the diagnosis of autism spectrum disorders. While different pipelines to analyze eye tracking data are available, increasing attention is now focused on the development of visual attention models using machine learning methods [46,47,48,49,50]. Since the diagnosis of autism is challenging and no biomarker is available [51], the development of computational models based on early abnormalities such as the differences in gaze processing might be of substantial help to improve and anticipate the diagnosis, thus, making it possible to initiate treatment at an earlier stage, when it is most effective [52]. Eye tracking measurements that might prove to be useful as early biomarkers include dysregulations in pupil dilation [53,54,55], changes in saccadic behavior, differences in gaze patterns during vision exposure to social stimuli [56,57,58] and analysis of scan paths or gaze patterns [59,60,61,62,63,64]. Some studies combined eye tracking data with other measurements such as resting-state EEG data [65,66,67]. Using this approach, Kang and colleagues showed a classification accuracy of up to 85.44% using a support vector machine (SVM) classifier to discriminate children aged from 3 to 6 with autism from controls [65]. Even higher performances were shown by another study conducted by Li and colleagues, using the three-layer Long Short-Term Memory (LSTM) network to discriminate 136 children with autism compared with 136 typically developing children based on gaze patterns during observations of 272 videos [68]. In this study, accuracy improved from 86.4% to 92.6% using LSTM compared to SVM [68]. Overall, results from these studies appear to be promising, although further research will be needed to evaluate their potential utility in the clinical setting and better assess the role of potential confounding factors that might affect gaze patterns (such as age, disease severity and duration, cognitive functioning and so on). Similarly, eye tracking measurements might be useful as prognostic markers [69] or to evaluate the efficacy of different types of interventions [70,71,72,73]. 

While we conducted a comprehensive analysis with no date and language restrictions, findings from our article must be interpreted in light of some limitations. First, our search might have missed some articles in which the terms included in our search strategy were mentioned in the main text but not in the title, abstract or keywords. In addition, our search might include some articles that mentioned the terms included in our search strategy but in which eye tracking experiments were not conducted (for instance reviews incorrectly classified by WOS as research articles, or articles in which new analytical methods were proposed). Despite these limitations, our analysis of large numbers of documents provides an updated and comprehensive picture of the use of eye tracking to study different medical conditions and highlights relevant trends with regards to investigated disorders and specific applications.

In conclusion, the eye tracking technique is increasingly being used in the study of different medical conditions. In autism spectrum disorders, eye tracking is widely used to evaluate eye movement abnormalities during face processing or looking at other social stimuli. Studies developing machine learning models provided promising evidence to support the utility of eye tracking measurements as biomarkers to discriminate patients with autism spectrum disorders from typically developing controls.

## Figures and Tables

**Table 1:** Country-specific scientific production.

Country	Articles	Single Country Publications	Multiple Country Publications	% of International Collaborations
USA	867	739	128	15%
UK	234	153	81	35%
Germany	178	122	56	31%
China	132	93	39	30%
Canada	108	84	24	22%
Australia	89	59	30	34%
France	85	69	16	19%
Italy	81	57	24	30%
Netherlands	73	53	20	27%
Japan	62	59	3	5%
Sweden	57	43	14	25%
Spain	48	32	16	33%
Poland	43	34	9	21%
Korea	38	35	3	8%
Switzerland	38	14	24	63%
Belgium	29	15	14	48%
India	29	23	6	21%
Brazil	23	15	8	35%
Austria	22	14	8	36%
Israel	22	11	11	50%
Denmark	15	10	5	33%
Finland	13	4	9	69%
Ireland	12	9	3	25%
Portugal	11	6	5	45%

The table reports, for each country, the total number of articles retrieved in the search, the articles with no international collaborations (single country publications), with international collaborations (multiple country publications) and the percentage of articles with international collaborations (international collaboration %). Only countries with at least ten articles retrieved with our search are included.

**Table 2:** Country-specific scientific production for articles focused on autism spectrum disorders.

Country	Articles	Single Country Publications	Multiple Country Publications	% of International Collaborations
USA	234	198	36	15%
UK	88	58	30	34%
China	52	36	16	31%
Australia	31	19	12	39%
France	31	24	7	23%
Sweden	23	16	7	30%
Germany	19	12	7	37%
Japan	18	16	2	11%
Italy	16	11	5	31%
Switzerland	15	2	13	87%
Belgium	13	7	6	46%
India	13	10	3	23%
Canada	12	7	5	42%
Netherlands	11	9	2	18%

The table reports, for each country, the total number of articles retrieved in the search and related to autism, the articles with no international collaborations (single country publications), with international collaborations (multiple country publications) and the percentage of articles with international collaborations (% of international collaboration). Only countries with at least ten articles retrieved with our search are included in the table.

**Table 3:** Twenty most cited articles focused on autism spectrum disorders.

Article	Cit	Cit/Year	Main Topic	Ref.
Dalton et al., 2005	954	56.12	Face processing	[27]
Jones et al., 2008	324	23.14	Face processing	[28]
Chawarska et al., 2013	265	29.44	Pictures with social scenes	[39]
Riby and Hacock, 2008	235	16.79	Pictures with social scenes	[40]
Pierce et al., 2011	209	19.00	Vision of images with geometric Patterns compared to social images	[44]
Speer et al., 2007	200	13.33	Face processing	[30]
Fletcher-Watson et al., 2009	196	15.08	Pictures with social scenes	[41]
Young et al., 2009	195	15.00	Face processing	[31]
Nacewicz et al., 2006	184	11.50	Discriminate facial expressions	[32]
Sasson et al., 2008	181	12.93	Social or nonsocial images	[42]
Van Der Geest et al., 2002	166	8.30	Face processing	[33]
Dalton et al., 2007	163	10.87	Face processing	[26]
Riby et al., 2009	162	12.46	Face processing	[29]
Neumann et al., 2006	156	9.75	Face processing	[34]
Elison et al., 2013	147	16.33	Oculomotor functioning and visual orienting	[45]
Nakano et al., 2010	147	12.25	Gaze patterns on social and nonsocial stimuli	[35]
Chawarska et al., 2012	146	14.60	Pictures with social scenes	[43]
Merlin et al., 2007	146	9.73	Videos with social scenes	[36]
Chawarska and Shic, 2009	145	11.15	Face processing	[37]
Wang et al., 2015	140	20.00	Face processing	[38]

The table shows, for the twenty most cited articles, the total number of citations (Cit), the number of citations per year (Cit/Year), the main topic of the article and the corresponding reference.

## Supplementary Materials

The following are available online at https://www.mdpi.com/article/10.3390/vision5040056/s1, Table S1: World collaboration network for articles investigating the use of eye tracking in different medical fields; Table S2: Most cited countries based on number of total citations; Table S3: Institutions with highest numbers of retrieved articles based on authors affiliations; Table S4: List of sources in which at least ten articles retrieved in the search were published; Table S5: World collaboration network for articles investigating the use of eye tracking in autism spectrum disorders; Table S6: Most cited countries based on number of total citations for articles focused on autism spectrum disorders; Table S7: Institutions with highest numbers of retrieved articles focused on autism spectrum disorders based on authors affiliations. Figure S1: Annual scientific production growth of all articles retrieved in our search; Figure S2: Annual scientific production growth of articles that applied eye tracking to the study of autism spectrum disorders; Figure S3: Country collaboration network of articles that applied eye tracking to the study of autism spectrum disorders.
