# Supplementary material for: Application of Eye Tracking Technology in Medicine: A Bibliometric Analysis
Source: Vision (Basel). 2021 Nov 11;5(4):56. doi: 10.3390/vision5040056 (PMC8628933; doi:10.3390/vision5040056)
Supplement: Supplementary file 1 [file vision-05-00056-s001.zip › Supplementary Tables.pdf]

**Table S1.** World collaboration network for articles investigating the use of eye tracking in different medical fields

| From | To | Frequency |
|---|---|---|
| Argentina | Colombia | 1 |
| Australia | Argentina | 1 |
| Australia | Brazil | 1 |
| Australia | Canada | 6 |
| Australia | Chile | 1 |
| Australia | Colombia | 1 |
| Australia | Italy | 6 |
| Australia | Mexico | 1 |
| Australia | Portugal | 1 |
| Australia | Qatar | 1 |
| Australia | Spain | 3 |
| Australia | Sweden | 17 |
| Austria | Russia | 1 |
| Belgium | Canada | 2 |
| Belgium | France | 6 |
| Belgium | Luxembourg | 2 |
| Belgium | Netherlands | 2 |
| Belgium | Poland | 1 |
| Belgium | Spain | 5 |
| Belgium | Switzerland | 3 |
| Belgium | UK | 3 |
| Belgium | USA | 10 |
| Brazil | Hungary | 1 |
| Canada | Austria | 2 |
| Canada | Brazil | 1 |
| Canada | Chile | 1 |
| Canada | Cyprus | 1 |
| Canada | Denmark | 1 |
| Canada | Mexico | 1 |
| Canada | Russia | 1 |
| Canada | Sweden | 2 |
| Canada | United Arab Emirates | 1 |
| Chile | Argentina | 1 |
| Chile | Colombia | 1 |
| China | Australia | 7 |
| China | Austria | 1 |
| China | Canada | 8 |
| China | France | 2 |
| China | Ireland | 1 |
| China | Japan | 6 |
| China | New Zealand | 1 |
| China | Singapore | 1 |
| China | Spain | 3 |
| China | Sweden | 1 |
| China | UK | 4 |

| | | |
|---|---|---|
| Denmark | Uruguay | 1 |
| Finland | Canada | 1 |
| Finland | Denmark | 1 |
| Finland | Ireland | 1 |
| Finland | Malawi | 2 |
| Finland | Russia | 1 |
| Finland | South africa | 2 |
| France | Brazil | 1 |
| France | Canada | 5 |
| France | Cyprus | 1 |
| France | Italy | 3 |
| France | Luxembourg | 1 |
| France | Malaysia | 2 |
| France | Mexico | 1 |
| France | Sweden | 8 |
| France | Switzerland | 14 |
| France | Tunisia | 1 |
| Germany | Australia | 8 |
| Germany | Austria | 9 |
| Germany | Canada | 6 |
| Germany | China | 3 |
| Germany | Cyprus | 1 |
| Germany | Finland | 1 |
| Germany | France | 3 |
| Germany | Greece | 1 |
| Germany | Israel | 1 |
| Germany | Italy | 11 |
| Germany | Japan | 1 |
| Germany | Korea | 2 |
| Germany | Poland | 3 |
| Germany | Russia | 1 |
| Germany | Slovakia | 1 |
| Germany | South africa | 2 |
| Germany | Spain | 2 |
| Germany | Sweden | 5 |
| Germany | Switzerland | 17 |
| Germany | UK | 25 |
| Greece | Turkey | 1 |
| India | Brazil | 1 |
| India | Canada | 1 |
| India | France | 1 |
| India | Ireland | 1 |
| India | New Zealand | 1 |
| Iran | Canada | 1 |
| Iran | Sweden | 2 |
| Israel | Canada | 1 |
| Israel | France | 1 |
| Israel | New Zealand | 1 |
| Italy | Argentina | 1 |

| | | |
|---|---|---|
| Italy | Austria | 1 |
| Italy | Brazil | 1 |
| Italy | Canada | 2 |
| Italy | Cyprus | 1 |
| Italy | Greece | 1 |
| Italy | Hungary | 1 |
| Italy | Iran | 2 |
| Italy | Ireland | 1 |
| Italy | Luxembourg | 1 |
| Italy | Norway | 1 |
| Italy | Portugal | 2 |
| Italy | Spain | 6 |
| Italy | Sweden | 4 |
| Japan | Austria | 1 |
| Japan | Bangladesh | 1 |
| Japan | France | 1 |
| Japan | Spain | 1 |
| Korea | Saudi Arabia | 1 |
| Luxembourg | Argentina | 2 |
| Luxembourg | Brazil | 1 |
| Luxembourg | Canada | 1 |
| Mexico | Spain | 1 |
| Netherlands | Australia | 4 |
| Netherlands | Austria | 2 |
| Netherlands | Canada | 3 |
| Netherlands | China | 1 |
| Netherlands | Cyprus | 1 |
| Netherlands | Denmark | 1 |
| Netherlands | France | 1 |
| Netherlands | Germany | 18 |
| Netherlands | India | 1 |
| Netherlands | Italy | 3 |
| Netherlands | Luxembourg | 1 |
| Netherlands | Mexico | 1 |
| Netherlands | Russia | 3 |
| Netherlands | South africa | 4 |
| Netherlands | Spain | 2 |
| Netherlands | Sweden | 5 |
| Netherlands | Switzerland | 4 |
| Netherlands | Turkey | 1 |
| Netherlands | UK | 23 |
| New Zealand | Austria | 1 |
| New Zealand | Uruguay | 1 |
| Poland | Hungary | 1 |
| Poland | Morocco | 1 |
| Portugal | Brazil | 1 |
| Portugal | Colombia | 1 |
| Portugal | Sweden | 3 |
| Qatar | Bangladesh | 1 |

| | | |
|---|---|---|
| Qatar | United Arab Emirates | 1 |
| Serbia | Slovenia | 2 |
| Singapore | Spain | 1 |
| South africa | Austria | 1 |
| South africa | Canada | 1 |
| South africa | Denmark | 1 |
| South africa | Sweden | 1 |
| Spain | Austria | 1 |
| Spain | Chile | 3 |
| Spain | Norway | 1 |
| Sweden | Austria | 2 |
| Sweden | Cyprus | 1 |
| Sweden | Denmark | 1 |
| Sweden | New Zealand | 1 |
| Sweden | Norway | 3 |
| Sweden | Poland | 2 |
| Sweden | Russia | 1 |
| Sweden | Spain | 1 |
| Switzerland | Austria | 1 |
| Switzerland | Brazil | 1 |
| Switzerland | Canada | 5 |
| Switzerland | Cyprus | 1 |
| Switzerland | Greece | 1 |
| Switzerland | Italy | 4 |
| Switzerland | Malaysia | 1 |
| Switzerland | Spain | 1 |
| Switzerland | Sweden | 5 |
| Switzerland | Turkey | 1 |
| Switzerland | United Arab Emirates | 1 |
| UK | Argentina | 2 |
| UK | Australia | 18 |
| UK | Austria | 2 |
| UK | Canada | 7 |
| UK | Chile | 2 |
| UK | Colombia | 1 |
| UK | Cyprus | 1 |
| UK | Denmark | 1 |
| UK | Finland | 4 |
| UK | France | 3 |
| UK | Georgia | 1 |
| UK | Greece | 1 |
| UK | Hungary | 1 |
| UK | India | 3 |
| UK | Iran | 2 |
| UK | Ireland | 6 |
| UK | Israel | 5 |
| UK | Italy | 14 |
| UK | Japan | 2 |
| UK | Malaysia | 2 |

| | | |
|---|---|---|
| UK | Mexico | 1 |
| UK | New Zealand | 4 |
| UK | Norway | 1 |
| UK | Poland | 3 |
| UK | Portugal | 5 |
| UK | Qatar | 1 |
| UK | Singapore | 1 |
| UK | South africa | 2 |
| UK | Spain | 6 |
| UK | Sweden | 7 |
| UK | Switzerland | 10 |
| UK | Turkey | 3 |
| UK | United Arab Emirates | 1 |
| USA | Argentina | 3 |
| USA | Australia | 24 |
| USA | Austria | 2 |
| USA | Bangladesh | 1 |
| USA | Brazil | 6 |
| USA | Canada | 47 |
| USA | Chile | 1 |
| USA | China | 31 |
| USA | Colombia | 1 |
| USA | Cyprus | 2 |
| USA | Denmark | 4 |
| USA | Finland | 1 |
| USA | France | 19 |
| USA | Germany | 29 |
| USA | Greece | 1 |
| USA | Hungary | 1 |
| USA | India | 4 |
| USA | Iran | 3 |
| USA | Ireland | 2 |
| USA | Israel | 18 |
| USA | Italy | 23 |
| USA | Japan | 8 |
| USA | Korea | 5 |
| USA | Kuwait | 1 |
| USA | Luxembourg | 1 |
| USA | Netherlands | 14 |
| USA | New Zealand | 3 |
| USA | Norway | 2 |
| USA | Peru | 3 |
| USA | Poland | 7 |
| USA | Portugal | 1 |
| USA | Qatar | 3 |
| USA | Russia | 2 |
| USA | Saudi Arabia | 1 |
| USA | Singapore | 3 |
| USA | South africa | 2 |

| | | |
|---|---|---|
| USA | Spain | 17 |
| USA | Sweden | 16 |
| USA | Switzerland | 16 |
| USA | Turkey | 1 |
| USA | United Arab Emirates | 2 |
| USA | UK | 47 |

The table shows, for each country, the number and type of international collaborations based on the articles retrieved in the search.

**Table S2.** Most cited countries based on number of total citations

| Country | Total Citations | Average Article Citations |
|---|---|---|
| USA | 23,434 | 27.029 |
| UK | 5,284 | 22.581 |
| Canada | 3,877 | 35.898 |
| Germany | 3,335 | 18.736 |
| France | 1,352 | 15.906 |
| Australia | 1,273 | 14.303 |
| Netherlands | 1,176 | 16.11 |
| China | 1,032 | 7.818 |
| Italy | 991 | 12.235 |
| Sweden | 769 | 13.491 |
| Japan | 578 | 9.323 |
| Spain | 530 | 11.042 |
| Switzerland | 499 | 13.132 |
| Denmark | 278 | 18.533 |
| Norway | 250 | 35.714 |
| Brazil | 243 | 10.565 |
| Austria | 201 | 9.136 |
| Korea | 201 | 5.289 |
| Israel | 193 | 8.773 |
| Poland | 186 | 4.326 |
| Belgium | 184 | 6.345 |
| Singapore | 154 | 38.5 |
| India | 125 | 4.31 |
| Ireland | 116 | 9.667 |
| Finland | 110 | 8.462 |
| Portugal | 110 | 10 |
| New Zealand | 95 | 11.875 |
| Argentina | 89 | 17.8 |
| Greece | 77 | 12.833 |
| United Arab Emirates | 47 | 9.4 |
| Uruguay | 47 | 15.667 |
| Qatar | 33 | 6.6 |
| Russia | 32 | 2.909 |
| Turkey | 23 | 2.556 |
| Malaysia | 18 | 9 |
| Peru | 18 | 9 |
| Iran | 17 | 1.889 |
| Cyprus | 14 | 7 |
| Hungary | 10 | 1.667 |
| Slovakia | 8 | 1 |
| Pakistan | 6 | 2 |
| Bangladesh | 5 | 2.5 |
| Mexico | 5 | 1 |
| South africa | 5 | 2.5 |
| Chile | 4 | 1 |
| Lithuania | 4 | 1.333 |

| | | |
|---|---|---|
| Colombia | 2 | 0.5 |
| Saudi Arabia | 2 | 1 |
| Thailand | 2 | 1 |
| Croatia | 1 | 0.5 |
| Romania | 1 | 0.333 |
| Senegal | 1 | 1 |
| Serbia | 1 | 0.5 |
| Bulgaria | 0 | 0 |
| Indonesia | 0 | 0 |
| Lebanon | 0 | 0 |
| Luxembourg | 0 | 0 |
| Slovenia | 0 | 0 |
| Sri Lanka | 0 | 0 |

The table shows, for each country, the number of total citations and the average number of citations per article.

**Table S3.** Institutions with highest numbers of retrieved articles based on authors' affiliations

| Affiliations | Articles |
|---|---|
| University of Toronto | 88 |
| Vanderbilt University | 83 |
| University of California Davis | 81 |
| Harvard University | 63 |
| Northwestern University | 61 |
| Kings College London | 59 |
| Duke University | 54 |
| University of California San Diego | 53 |
| La Trobe University | 50 |
| University Iowa | 48 |
| University North Carolina | 48 |
| Uppsala University | 48 |
| Emory University | 47 |
| Karolinska Institute | 47 |
| University of Pittsburgh | 46 |
| Yale University | 45 |
| Macquarie University | 43 |
| University of Penn | 43 |
| University of Washington | 43 |
| Stanford University | 42 |
| University of Wisconsin | 42 |
| Boston University | 39 |
| University of Illinois | 37 |
| Johns Hopkins University | 36 |
| University of California Los Angeles | 36 |
| University Minnesota | 35 |
| Harvard Medical School | 34 |
| University of Utrecht | 34 |
| Peking University | 33 |
| University of Tubingen | 33 |
| Columbia University | 32 |
| University of Oxford | 31 |
| Curtin University | 30 |
| University of Cambridge | 30 |
| McGill University | 29 |
| Tel Aviv University | 29 |
| University of Geneva | 29 |
| Penn State University | 28 |
| Indiana University | 27 |
| Queens University | 27 |
| University of Edinburgh | 27 |
| Washington University | 27 |
| Maastricht University | 26 |
| Radboud University Nijmegen | 26 |
| School of Medicine | 26 |
| University of California San Francisco | 26 |

| | |
|---|---|
| University of Kansas | 26 |
| Brown University | 25 |
| Sun Yat-Sen University | 25 |
| Newcastle University | 24 |
| University of Barcelona | 24 |
| University of British Columbia | 24 |
| University of London | 24 |
| University of Melbourne | 23 |
| University of Texas Dallas | 23 |
| Albert Einstein College of Medicine | 22 |
| Purdue University | 22 |
| University of Leipzig | 22 |
| University of Rochester | 22 |
| University of Siena | 22 |
| University of Lubeck | 21 |
| University of Massachusetts | 21 |
| Heidelberg University | 20 |
| University of Maryland | 20 |
| University of Bern | 19 |
| University of Fukui | 19 |
| University of Ghent | 19 |
| Baylor College of Medicine | 18 |
| University of California Berkeley | 18 |
| University of Groningen | 18 |
| University of Kentucky | 18 |
| University of Texas Austin | 18 |
| University of Zurich | 18 |
| Lund University | 17 |
| Massachusetts General Hospital | 17 |
| Medical University Vienna | 17 |
| Northeastern University | 17 |
| Osaka University | 17 |
| University of Alberta | 17 |
| University of Coimbra | 17 |
| University of Munster | 17 |
| University of Nottingham | 17 |
| Beijing Normal University | 16 |
| Jonkoping University | 16 |
| Leiden University | 16 |
| Monash University | 16 |
| Rush University | 16 |
| University of Munich | 16 |
| University of Western Ontario | 16 |
| Ewha Womans University | 15 |
| Florida State University | 15 |
| University of Amsterdam | 15 |
| University of Calgary | 15 |
| University of Messina | 15 |
| University of Milan | 15 |

| | |
|---|---|
| University of Oslo | 15 |
| University of Sheffield | 15 |
| University of South Carolina | 15 |
| University of Sydney | 15 |
| University of Tokyo | 15 |
| University of Western Australia | 15 |
| Carnegie Mellon University | 14 |
| Linkoping University | 14 |
| Ohio University | 14 |
| Temple University | 14 |
| University of Alabama Birmingham | 14 |
| University of Freiburg | 14 |
| University of Lancaster | 14 |
| University of Reading | 14 |
| University of Southampton | 14 |
| Virginia Commonwealth University | 14 |
| Birkbeck University London | 13 |
| Capital Medical University | 13 |
| Chinese University Hong Kong | 13 |
| City University London | 13 |
| Dept Neurosci | 13 |
| Drexel University | 13 |
| Gdańsk University of Technology | 13 |
| Katholieke University Leuven | 13 |
| University of Chicago | 13 |
| University of Gothenburg | 13 |
| UT Health Science Center at Houston | 13 |
| York University | 13 |
| Humboldt University | 12 |
| Institute of Neurology | 12 |
| Ohio State University | 12 |
| University of Complutense Madrid | 12 |
| University of Copenhagen | 12 |
| University of Houston | 12 |
| University of Sao Paulo | 12 |
| Yale School of Medicine | 12 |
| Anhui Medical University | 11 |
| Bar Ilan University | 11 |
| Cardiff University | 11 |
| Center for Psychiatric Research | 11 |
| Ludwig Maximilians University Munchen | 11 |
| McLean Hospital | 11 |
| Moss Rehabilitation Research Insistute | 11 |
| Paris | 11 |
| Queens University of Belfast | 11 |
| University of Cincinnati | 11 |
| University of Connecticut | 11 |
| University of Georgia | 11 |
| University of Manchester | 11 |

| | |
|---|---|
| West Virginia University | 11 |
| Aix Marseille University | 10 |
| Cornell University | 10 |
| Haskins Laboratories | 10 |
| Hong Kong Polytech University | 10 |
| LuEsther T. Mertz Retinal Research Center | 10 |
| Medical University Hospital of Tubingen | 10 |
| Seattle Childrens Research Instistute | 10 |
| Seoul Natl University | 10 |
| Simon Fraser University | 10 |
| University of Cape Town | 10 |
| University of Cent Florida | 10 |
| University of Chile | 10 |
| University of Colorado | 10 |
| University of Durham | 10 |
| University of Lille | 10 |
| University of Missouri | 10 |
| University Prebiteriana Mackenzie | 10 |

The table shows the number of articles per institution based on authors' affiliations. Institutions with at least 10 published articles are reported.

**Table S4.** List of sources in which at least 10 articles retrieved in the search were published

| Sources | Articles |
|---|---|
| Journal of Autism and Developmental Disorders | 104 |
| PLOS ONE | 65 |
| Autism Research | 51 |
| Frontiers in Psychology | 49 |
| Psychiatry Research | 36 |
| Schizophrenia Research | 35 |
| Research in Autism Spectrum Disorders | 32 |
| Biological Psychiatry | 28 |
| Neuropsychologia | 25 |
| Scientific Reports | 25 |
| Investigative Ophthalmology & Visual Science | 23 |
| Frontiers in Psychiatry | 22 |
| Journal of Abnormal Psychology | 22 |
| American Journal of Psychiatry | 21 |
| Frontiers in Human Neuroscience | 21 |
| Molecular Autism | 21 |
| Autism | 20 |
| Journal of Neurodevelopmental Disorders | 18 |
| Cognition | 17 |
| Journal of Speech Language and Hearing Research | 17 |
| Behaviour Research and Therapy | 16 |
| Cognition & Emotion | 16 |
| Experimental Brain Research | 16 |
| Research in Developmental Disabilities | 16 |
| Journal of Behavior Therapy and Experimental Psychiatry | 15 |
| Archives of General Psychiatry | 14 |
| Cognitive Therapy and Research | 14 |
| Communication Sciences and Disorders-Csd | 13 |
| Journal of Affective Disorders | 13 |
| Retina-The Journal of Retinal and Vitreous Diseases | 13 |
| Frontiers in Neuroscience | 12 |
| Journal of Alzheimers Disease | 12 |
| Journal of Psychiatric Research | 12 |
| Developmental Psychology | 11 |
| Developmental Science | 11 |
| European Archives of Psychiatry and Clinical Neuroscience | 11 |
| Frontiers in Neurology | 11 |
| International Journal of Eating Disorders | 11 |
| Journal of Anxiety Disorders | 11 |
| Journal of Child Psychology and Psychiatry | 11 |
| Biological Psychology | 10 |
| Infant Behavior & Development | 10 |
| Journal of Experimental Child Psychology | 10 |
| Journal of Neuroscience Methods | 10 |
| Language Cognition and Neuroscience | 10 |
| Quarterly Journal of Experimental Psychology | 10 |

The table shows the number of articles per scientific journal.

**Table S5.** World collaboration network for articles investigating the use of eye tracking in autism spectrum disorders

| From | To | Frequency |
|---|---|---|
| Argentina | Luxembourg | 1 |
| Australia | Canada | 2 |
| Australia | Germany | 1 |
| Australia | Italy | 1 |
| Australia | Mexico | 1 |
| Australia | Qatar | 1 |
| Australia | Sweden | 12 |
| Australia | UK | 6 |
| Austria | New Zealand | 1 |
| Belgium | France | 2 |
| Belgium | Switzerland | 1 |
| Belgium | UK | 1 |
| Belgium | USA | 6 |
| Canada | Chile | 1 |
| Canada | Cyprus | 1 |
| China | Canada | 7 |
| China | Sweden | 1 |
| France | Canada | 2 |
| France | China | 1 |
| France | Cyprus | 1 |
| France | Germany | 2 |
| France | Israel | 1 |
| France | Italy | 3 |
| France | Netherlands | 1 |
| France | Sweden | 7 |
| France | Switzerland | 12 |
| France | UK | 2 |
| Germany | Austria | 1 |
| Germany | Canada | 1 |
| Germany | Cyprus | 1 |
| Germany | Greece | 1 |
| Germany | Netherlands | 2 |
| Germany | Sweden | 2 |
| India | UK | 2 |
| India | USA | 4 |
| Iran | Canada | 1 |
| Iran | Sweden | 1 |
| Italy | Argentina | 1 |
| Italy | Canada | 1 |
| Italy | Cyprus | 1 |
| Italy | Germany | 3 |
| Italy | Greece | 1 |
| Italy | Luxembourg | 1 |
| Italy | Netherlands | 1 |
| Italy | Norway | 1 |

| | | |
|---|---|---|
| Italy | Spain | 2 |
| Italy | Sweden | 2 |
| Japan | Bangladesh | 1 |
| Netherlands | Canada | 1 |
| Netherlands | Cyprus | 1 |
| Qatar | United Arab Emirates | 1 |
| Spain | Norway | 1 |
| Spain | Sweden | 1 |
| Sweden | Austria | 1 |
| Sweden | Canada | 1 |
| Sweden | Cyprus | 1 |
| Sweden | Netherlands | 1 |
| Sweden | New Zealand | 1 |
| Sweden | Norway | 1 |
| Sweden | Poland | 1 |
| Switzerland | Brazil | 1 |
| Switzerland | Canada | 2 |
| Switzerland | Cyprus | 1 |
| Switzerland | Germany | 4 |
| Switzerland | Italy | 1 |
| Switzerland | Malaysia | 1 |
| Switzerland | Netherlands | 1 |
| Switzerland | Sweden | 4 |
| Switzerland | United Arab Emirates | 1 |
| Switzerland | UK | 3 |
| UK | Canada | 3 |
| UK | Cyprus | 1 |
| UK | Finland | 2 |
| UK | Germany | 4 |
| UK | Greece | 1 |
| UK | Hungary | 1 |
| UK | Ireland | 4 |
| UK | Italy | 7 |
| UK | Japan | 2 |
| UK | Malaysia | 2 |
| UK | Mexico | 1 |
| UK | Netherlands | 2 |
| UK | New Zealand | 1 |
| UK | Poland | 2 |
| UK | Portugal | 1 |
| UK | Qatar | 1 |
| UK | Spain | 1 |
| UK | Sweden | 4 |
| UK | Turkey | 3 |
| UK | United Arab Emirates | 1 |
| USA | Australia | 12 |
| USA | Canada | 15 |
| USA | China | 17 |
| USA | Cyprus | 1 |

| | | |
|---|---|---|
| USA | France | 9 |
| USA | Germany | 5 |
| USA | Iran | 1 |
| USA | Israel | 1 |
| USA | Italy | 8 |
| USA | Japan | 1 |
| USA | Korea | 1 |
| USA | Netherlands | 1 |
| USA | Norway | 2 |
| USA | Peru | 3 |
| USA | Poland | 1 |
| USA | Qatar | 2 |
| USA | Singapore | 1 |
| USA | Sweden | 11 |
| USA | Switzerland | 12 |
| USA | Turkey | 1 |
| USA | United Arab Emirates | 2 |
| USA | UK | 15 |

The table shows, for each country, the number and type of international collaborations based on the articles retrieved in the search and related to autism.

**Table S6.** Most cited countries based on number of total citations for articles focused on autism spectrum disorders

| Country | Total Citations | Average Article Citations |
|---|---|---|
| USA | 7,671 | 32.78 |
| UK | 2,326 | 26.43 |
| Sweden | 507 | 22.04 |
| Australia | 487 | 15.71 |
| China | 438 | 8.42 |
| France | 395 | 12.74 |
| Netherlands | 384 | 34.91 |
| Japan | 324 | 18.00 |
| Germany | 288 | 15.16 |
| Canada | 237 | 19.75 |
| Switzerland | 196 | 13.07 |
| Singapore | 143 | 71.50 |
| Italy | 112 | 7.00 |
| Denmark | 100 | 33.33 |
| India | 100 | 7.69 |
| Belgium | 76 | 5.85 |
| Ireland | 50 | 16.67 |
| Brazil | 41 | 8.20 |
| Qatar | 27 | 6.75 |
| Finland | 25 | 5.00 |
| Poland | 20 | 4.00 |
| Portugal | 20 | 10.00 |
| Spain | 20 | 2.86 |
| Malaysia | 18 | 18.00 |
| Peru | 18 | 9.00 |
| Norway | 13 | 6.50 |
| Bangladesh | 5 | 5.00 |
| Hungary | 4 | 2.00 |
| Israel | 3 | 1.00 |
| Turkey | 2 | 0.67 |
| Iran | 1 | 0.33 |
| Saudi Arabia | 1 | 1.00 |
| Austria | 0 | 0.00 |
| Bulgaria | 0 | 0.00 |
| Lithuania | 0 | 0.00 |
| Russia | 0 | 0.00 |
| United Arab Emirates | 0 | 0.00 |

The table shows, for each country, the number of total citations and the average number of citations per article.

**Table S7.** Institutions with highest numbers of retrieved articles focused on autism spectrum disorders based on authors' affiliations

| Affiliations | Articles |
|---|---|
| Vanderbilt University | 54 |
| La Trobe University | 45 |
| Duke University | 44 |
| University of North Carolina | 42 |
| University of Washington | 35 |
| University of California Davis | 31 |
| Yale University | 30 |
| Kings College London | 29 |
| Curtin University | 26 |
| Karolinska Institute | 24 |
| Uppsala University | 22 |
| Peking University | 21 |
| University of London | 20 |
| University of Wisconsin | 20 |
| Emory University | 19 |
| Sun Yat-Sen University | 18 |
| University of Cambridge | 18 |
| University of Fukui | 16 |
| Harvard University | 15 |
| University California San Diego | 15 |
| University of Nottingham | 15 |
| University of Penn | 15 |
| University of Texas Dallas | 15 |
| Boston University | 14 |
| Macquarie University | 14 |
| Stanford University | 14 |
| University of Geneva | 14 |
| Jonkoping University | 13 |
| University of California Los Angeles | 12 |
| University of Gothenburg | 11 |
| Brown University | 10 |
| Harvard Medical School | 10 |
| Katholieke University Leuven | 10 |
| Linkoping University | 10 |
| McGill University | 10 |
| Penn State University | 10 |
| Seattle Childrens Research Insistute | 10 |
| University of Coimbra | 10 |
| University of Kansas | 10 |
| University of Massachusetts | 10 |
| University of Utrecht | 10 |
| Yale School of Medicine | 10 |

The table shows the number of articles per institution based on authors' affiliations. Institutions with at least 10 published articles are reported.
