# Supplementary figures and images for: Application of Eye Tracking Technology in Medicine: A Bibliometric Analysis
Source: Vision (Basel). 2021 Nov 11;5(4):56. doi: 10.3390/vision5040056 (PMC8628933; doi:10.3390/vision5040056)

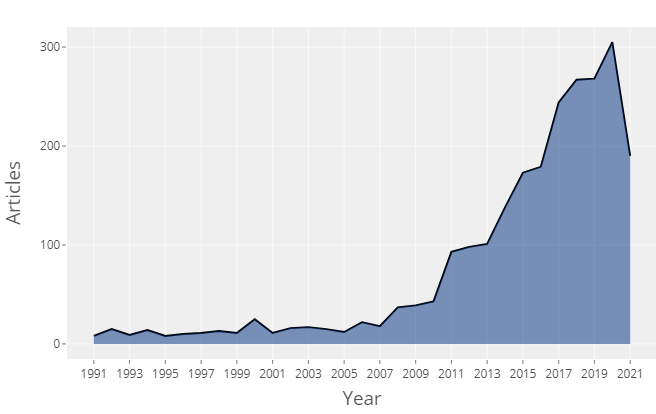

Supplement: Supplementary file 1 [file vision-05-00056-s001.zip › Figure S1.png]

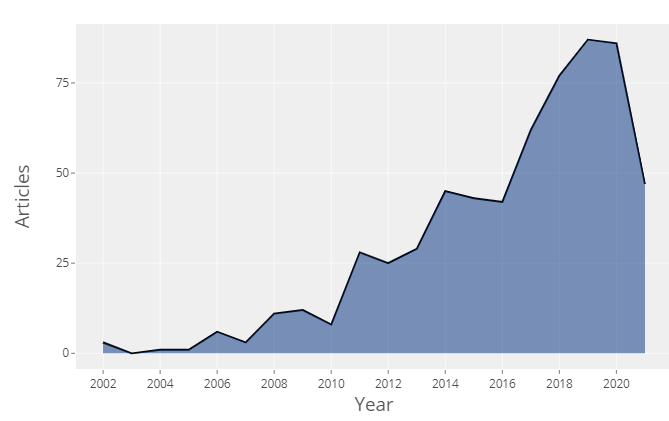

Supplement: Supplementary file 1 [file vision-05-00056-s001.zip › Figure S2.png]

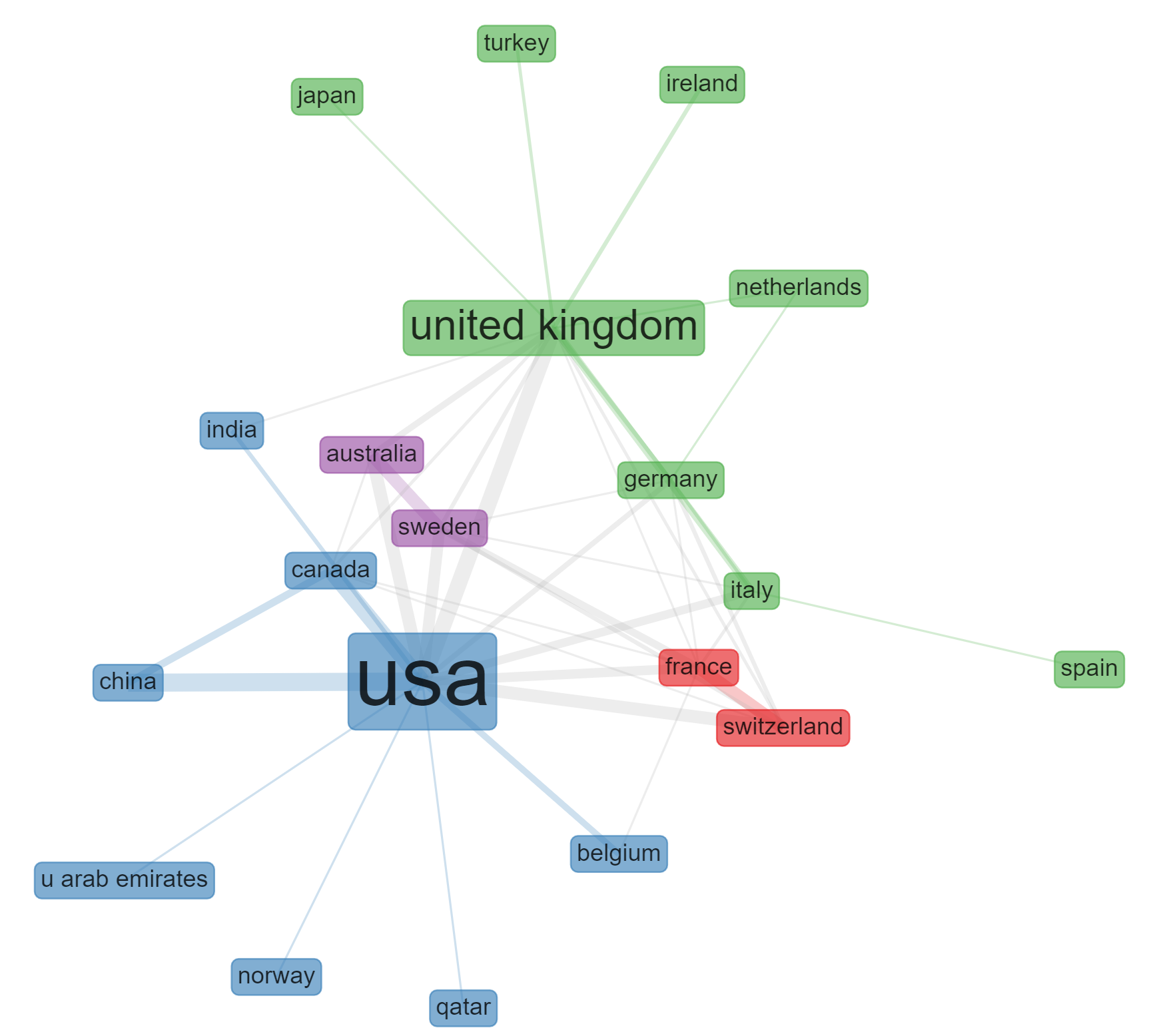

Supplement: Supplementary file 1 [file vision-05-00056-s001.zip › Figure S3.png]
